# Effect of Low Gravity Solids on Weak Gel Structure and the Performance of Oil-Based Drilling Fluids

**DOI:** 10.3390/gels9090729

**Published:** 2023-09-08

**Authors:** Haokun Shen, Jinsheng Sun, Kaihe Lv, Meichun Li, Yuan Geng, Zheng Yang, Xianbin Huang, Hongyan Du, Muhammad Arqam Khan

**Affiliations:** 1 School of Petroleum Engineering, China University of Petroleum, Qingdao 266580, China; b20020017@s.upc.edu.cn (H.S.); mli@upc.edu.cn (M.L.);; 2 Key Laboratory of Unconventional Oil & Gas, Development Ministry of Education, Qingdao 266580, China; 3 CNPC Engineering Technology R&D Company Ltd., Beijing 102206, China; gengyuan0421waiting@126.com (Y.G.); yangzhdr@cnpc.com.cn (Z.Y.); 4 Co-Innovation Center of Efficient Processing and Utilization of Forest Resources, College of Materials Science and Engineering, Nanjing Forestry University, Nanjing 210037, China; 5 Department of Petroleum Engineering, NED University of Engineering & Technology, Karachi 395007, Pakistan

**Keywords:** low gravity solids, oil-based drilling fluid, weak gel structure, rheology

## Abstract

Drilling cuttings from the rock formation generated during the drilling process are generally smashed to fine particles through hydraulic cutting and grinding using a drilling tool, and then are mixed with the drilling fluid during circulation. However, some of these particles are too small and light to be effectively removed from the drilling fluid via solids-control equipment. These small and light solids are referred to as low gravity solids (LGSs). This work aimed to investigate the effect of LGSs on the performance of oil-based drilling fluid (OBDF), such as the rheological properties, high-temperature and high-pressure filtration loss, emulsion stability, and filter cake quality. The results show that when the content of LGSs reached or even exceeded the solid capacity limit of the OBDF, the rheological parameters including the plastic viscosity, gel strength, and thixotropy of OBDF increased significantly. Furthermore, the filtration of OBDF increases, the filter cake becomes thicker, the friction resistance becomes larger, and the stability of emulsion of OBDF also decreases significantly when the concentration of LGSs reached the solid capacity limit of OBDF (6–9 wt% commonly). It was also found that LGSs with a smaller particle size had a more pronounced negative impact on the drilling fluid performance. This work provides guidance for understanding the impact mechanism of LGSs on drilling fluid performance and regulating the performance of OBDF.

## 1. Introduction

Oil-based drilling fluid (OBDF) refers to a non-aqueous solvent drilling fluid that uses oil (such as diesel, mineral oil, and synthetic oil) as the continuous phase, as opposed to water-based drilling fluid [1,2,3]. OBDF is widely used in complex well sections such as deep wells, ultra-deep wells, highly deviated wells, and horizontal wells due to its excellent inhibition, lubrication, and high temperature resistance [4].

During the drilling process, drilling cuttings composed of rocks and low-quality clay are mixed into OBDF. In addition, the commercial solids (such as barite, calcium carbonate, and organic clay) intentionally added to OBDF, drilling cuttings represent a significant source of solid particles. The volume of rock debris or drilling solids removed from the borehole is enormous and can be estimated using the following formula:

(1) $$V_{s}=\pi (1-\varphi )r^{2}\times (ROP)$$

where *V_s_* is the volume of rock fragments entering the OBDF, *φ* is the average porosity of the formation, *r* is the bit radius, and *ROP* is an abbreviation for the rate of penetration. Usually, up to 100,000 pounds of rock debris could be carried with the drilling fluid every day.

The primary function of a drilling fluid is to carry the drilling cuttings generated by the drill bit during the drilling process. This is achieved via circulation, where the drilling fluid carries the cuttings to the surface, and they are subsequently removed using solid-control equipment. This ensures continuous drilling and the advancement of the wellbore [5,6]. Furthermore, when the drilling fluid circulation is halted, an efficient drilling fluid should be able to suspend the drilling cuttings in the wellbore to prevent their settling. However, sometimes because of the reduced rock-carrying capacity of the drilling fluid in horizontal sections and highly deviated wells, some drilling cuttings may retain in the wellbore and undergo repeated squeezing and grinding by the drill bit and drilling tools, resulting in a significant reduction in the particle size of these cuttings [7]. In addition, under high shear rates, the friction between the drilling fluid, drilling chips, as well as among the chips themselves can cause the drilling chips to disintegrate into fine particles [8]. When the particle size of these fine particles is lower than the separation limit of the solid-control equipment, or if there are limitations in the disposal time and separation efficiency of the solid-control equipment, these fine particles, known as low gravity solids (LGSs), will be retained in OBDF [9]. Generally, the presence of a small amount of LGSs in OBDF is not considered detrimental. In fact, some studies have suggested that a small amount of LGSs is beneficial for improving the suspension capacity of OBDF. Fagundes [10] et al. used gamma ray attenuation technology to detect the function of LGS concentration in oil-based drilling fluids over time. The results showed that after 90 days, a clear liquid region was formed between 16 and 22 cm from the bottom of the pipe. This area forms between 7 and 80 days, with a maximum solid concentration of approximately 9%. And, as the concentration of LGS increases, the fluid resistance of solid sedimentation also increases.

However, due to the high preparation cost of OBDF, the method of multi-well circulation is applied to reduce the overall cost of drilling fluid. Recycling drilling fluid and improper solid-control methods could lead to a gradual increase in the LGSs’ content. Because it is impossible to entirely remove all of the drilling particles from the drilling fluid, they are considered persistent pollutants in the fluid system, which highlights the significance of this study. The degree of pollution caused by these drilling solids depends largely on their content, particle size, and type [11]. Up to now, although there are some literature investigating the different drilling fluids’ basis, composition, and the LGSs, where most of them primarily focus on the solid-control equipment and its efficiency, rather than the impact of LGSs on the rheological properties, filtration, and emulsion stability of drilling fluids [12,13,14]. Derrick et al. [15] investigated the effect of drilled solids on the drilling rate and drilling performance. Experiments show that the ES drops in half as the drilled solid (bentonite) concentration increases from 0 to 75 lb/bbl. The high temperature and high pressure fluid loss test produced thicker filter cake and a doubled fluid loss, while the YP quadruples as the bentonite concentration increases.

Since the LGSs are an inevitable product during the drilling process, understanding the effect of LGSs on the performance of OBDF is crucial for adjusting the drilling fluid properties and mitigating the deterioration of the drilling fluid performance. Furthermore, it is necessary to monitor closely the content of LGS in OBDF and maintain the drilling fluid in a timely manner during the drilling process.

With this in mind, the objective of this study is to evaluate the impact of LGSs on various important properties of OBDF, including rheology, high-temperature and high-pressure (HTHP) filtration, emulsion stability, and filter cake quality. Firstly, a new method was designed to separate LGSs from waste OBDF. Then, the impact of LGSs on the rheological properties of OBDF is evaluated through adding the separated LGSs into the freshly prepared OBDF. Differently from previous papers, this study further investigated the influence of LGS on the rheological properties of drilling fluids via steady-state shear experiments, the recovery rate of drilling fluid structures at low shear rates, and the thixotropy ring method. Furthermore, to gain a more comprehensive understanding of the impact of LGS on filtration, emulsion stability, and the other properties of drilling fluids, the filtration test, mud cake property, and emulsion stability test were performed.

## 2. Results and Discussion

**Figure 1:**
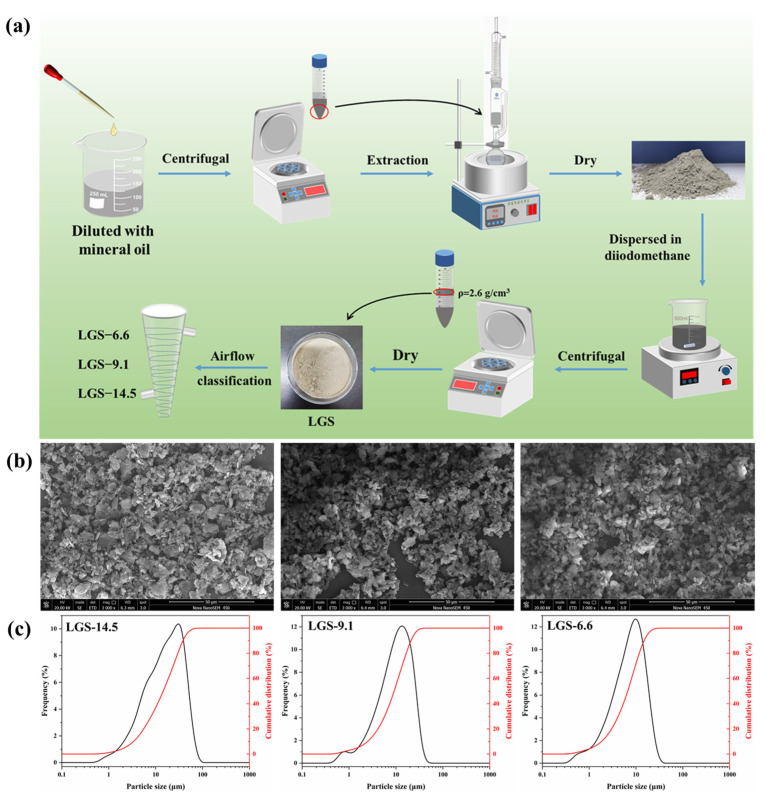
Separation process and morphology of LCSs from waste OBDF: (**a**) separation scheme, (**b**) SEM images, and (**c**) particle size distribution of LGSs.

Figure 1 schematically illustrates the separation process of LGSs. To separate the solids in the waste OBDF, the waste OBDF sample was initially diluted with mineral oil, and afterwards separated out via high-speed centrifugation. Then, diiodomethane was used as the dispersion medium to separate LGSs from the weighting material, taking advantage of the density difference between LGSs (2.6 g/cm^3^) and the weighting material (4.2 g/cm^3^) in the drilling fluid. An airflow classifier (JZF-100, Sichuan Juzi powder Equipment Co., Ltd., Mianyang, China) was further used to classify LGSs based on their particle sizes. In the airflow classifier, LGSs were subject to the impact and disturbance of the airflow. Due to the different particle sizes of LGSs, the motion state of LGSs changed accordingly. In this work, LGSs were divided into three categories according to their median particle size (D_50_), i.e., LGS-14.5, LGS-9.1, and LGS-6.6.

As shown in Figure 1b, SEM revealed the morphology of the separated LGSs, showing an irregular, spherical, and sheet-like shape. In addition, the edges of the particles are relatively smooth, which is caused by the fluid washing in the drilling fluid. Particle size distribution curves (Figure 1c) of the three types of LGSs indicated that the particle size distribution of the three LGSs are similar, but there is a new peak at around 1 μm for LGS-9.1 and LGS-6.6. Table 1 shows detailed particle size information for the three types of LGSs. The weight specific surface areas of LGS-14.5, LGS-9.1, and LGS-6.6 are 801.844 m^2^/kg, 1185.754 m^2^/kg, and 1579.346 m^2^/kg, respectively, which is consistent with the results of the particle size reduction.

**Figure 2:**
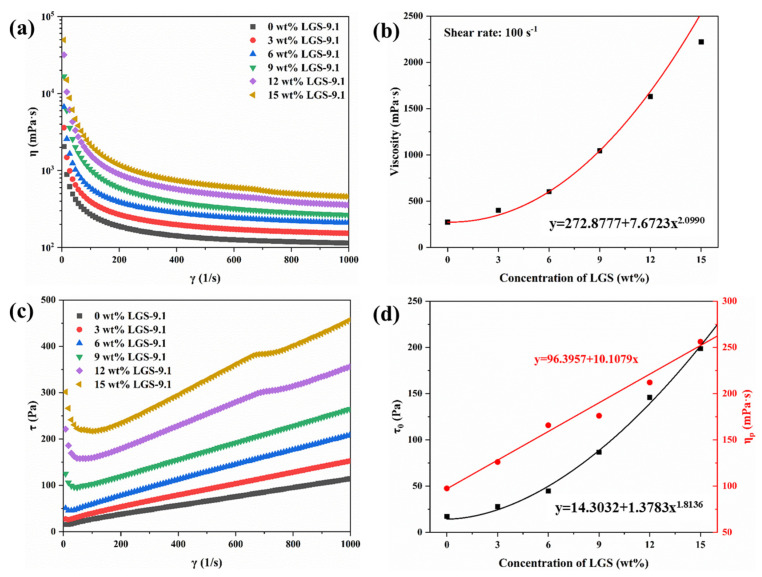
Effect of LGSs concentration on the rheology of OBDF: (**a**) Viscosity as a function of shear rate. (**b**) The viscosity at a shear rate of 100 s^−1^. (**c**) Shear stress as a function of shear rate. (**d**) Rheological parameters of the Bingham model and fitting results.

The organic clay and barite added to OBDF are usually irregularly shaped particles that easily interact with other particles and droplets, creating a spatial network structure with a certain gel strength [19]. As shown in Figure 2a, the network structure present in drilling fluids is disrupted at high shear rates, and all samples exhibit a typical shear-thinning behavior (i.e., viscosity decreases with the increasing shear rates). The viscosity of drilling fluid at a fixed shear rate (100 s^−1^) is positively correlated with the concentration of LGSs (Figure 2b). For example, for OBDF containing 3 wt%, 6 wt%, 9 wt%, 12 wt%, and 15 wt%, the viscosity at 100 s^−1^ is 401.02 mPa·s, 602.69 mPa·s, 1045.36 mPa·s, 1630.28 mPa·s, and 2221.45 mPa·s, respectively. This is because LGSs can form a stronger spatial network structure under high load, resulting in higher viscosities. The results of fitting the viscosity at 100 s^−1^ to the concentration of LGSs indicate that the relationship between viscosity and LGSs is an exponential function, suggesting that LGSs have a more pronounced impact on viscosity at high concentrations.

As shown in Figure 2c, the flow curve of the sample is not a horizontal straight line, indicating that the fluid is non-Newtonian. The non-zero intercepts indicated that the applied shear stress must exceed a certain minimum value before flow can begin, exhibiting the characteristics of a plastic fluid. For OBDF, the Bingham model was used commonly to fit the shear stress as a function of the shear rate. According to the fitted yield stress values (τ_0_) of 17.23 Pa, 27.66 Pa, 44.70 Pa, 86.56 Pa, 145.82 Pa, and 198.62 Pa for drilling fluids with the LGSs’ concentrations of 0 wt%, 3 wt%, 6 wt%, 9 wt%, 12 wt%, and 15 wt%, respectively. The gel strength of the drilling fluid is also positively correlated with the amount of LGSs added. Similarly, the fitted plastic viscosity values (η_p_) of 97.44 mPa·s, 126.01 mPa·s, 165.73 mPa·s, 175.99 mPa·s, 212.00 mPa·s, and 256.06 mPa·s for drilling fluids with the LGSs’ concentrations of 0 wt%, 3 wt%, 6 wt%, 9 wt%, 12 wt%, and 15 wt%, respectively, indicate a positive correlation between the frictional resistance in the drilling fluid and the amount of LGSs added. In order to reduce the annular circulation pressure loss, reduce the bottom hole back pressure, and increase the mechanical drilling speed, regardless of the drilling fluid density or other requirements of the downhole working conditions, it is better to have a smaller η_p_ because of the annular circulation pressure loss Δp∞η_p_. τ_0_ is one of the most obvious rheological parameters that determine the fluctuating pressure. A large number of studies abroad have shown that only changing the ratio from 30 Pa to 50 Pa significantly increases the bottom fluctuation pressure, and the annular pressure loss Δp is also proportional to τ_0_. Therefore, the maximum allowable limit set by foreign countries is 50 Pa. Moreover, research has shown that for the benefit of wellbore purification, the minimum limit of τ_0_ is 15~20 Pa, and the density of the high-density drilling fluid helps to improve the rock carrying efficiency without requiring too high rheological parameters of the carrier. Figure 2d shows the results of fitting the τ_0_ and η_p_ with the LGSs’ concentration. It was observed that τ_0_ satisfied an exponential equation with the LGSs’ concentration, while η_p_ satisfied a linear equation. This is because the yield stress reflects the interaction force between the particles and the drilling fluid treatment agents during laminar flow of fluid. This force significantly increases with the increase in solid particles, resulting in a geometrically amplified strength of the spatial grid structure. The plastic viscosity reflects the internal friction between particles, droplets, and liquids in the drilling fluid. It is mainly related to the solid content or particle number. The viscosity of the suspension in consistent units is given via the Krieger–Dougherty equation [20]:

(2) $$\eta =\eta_{0}(1-\frac{\varphi }{\varphi_{m}}{)}^{-[\eta ]\varphi_{m}}$$

where *η* is the viscosity of suspension, *η*_0_ is the viscosity of the medium, *φ* is the volume fraction of the solid particles, *φ_m_* is the maximum volume fraction of the solid particles that can be dispersed and fully wetted in a continuous phase, and [η] is the intrinsic viscosity of the medium.

Thixotropy is also an important rheological parameter of drilling fluid. Thixotropy is the structural degradation caused by the breakage of flocculated or interconnected particles during shearing. When the shear stress is eliminated, the microstructure of drilling fluid will be reconstructed and ultimately restored to its original state [21,22]. Drilling fluid should have proper thixotropy; that is, it should exhibit a short recovery time and low gel strength. These characteristics are necessary for improving the penetration rate, reducing pump pressure, efficient rock carrying, and suspending weighting materials [23]. The common methods used to evaluate fluid thixotropy mainly include the three-stage rheological test and the thixotropic loop method.

**Figure 3:**
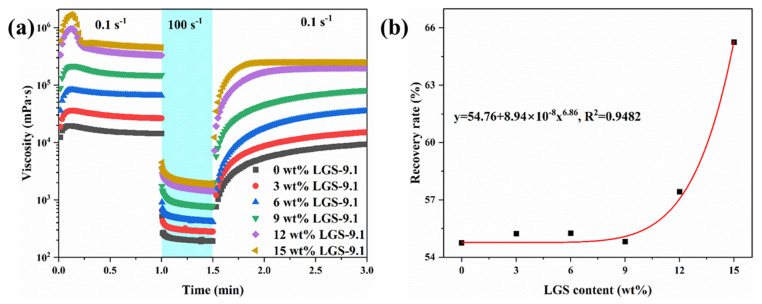
(**a**) Viscosity progression over time when applying low (0.1 s^−1^) and high (100 s^−1^) shear rates alternatively; (**b**) the recovery rate of viscosity when the shear rate decreased again to 0.1 s and sustained 1.5 min. The recovery rate is the quotient of viscosity at 1 min and viscosity at 3 min.

As shown in Figure 3a, the viscosity of the drilling fluid remained at a high level when the shear rate was 0.1 s^−1^, indicating the presence of a high strength gel structure in drilling fluid. However, when the shear rate increased to 100 s^−1^, the viscosity of the drilling fluid gradually decreased and stabilized, indicating that the structure in the drilling fluid was damaged and changed to a sol state. Furthermore, when the shear rate restored to 0.1 s^−1^, the viscosity of the drilling fluid gradually recovered to a high viscosity, indicating a gradual restoration of the drilling fluid structure. In addition, as the LGSs’ content increased, the viscosity of the drilling fluid also increased. When the LGSs’ content in the drilling fluid was increased from 0 wt% to 15 wt%, the viscosity of the drilling fluid increased by nearly 100 times at 0.1 s^−1^ and nearly 10 times at 100 s^−1^. Moreover, when the viscosity of the drilling fluid was decreased again to a low shear rate, the recovery rate of viscosity was faster, indicating that the introduction of LGSs enhanced the structural recovery ability of the drilling fluid at low shear rates. The results of structure recovery show that (Figure 3b), with the increase in low-density solid content, the structure recovery rate of drilling fluid increases significantly after stopping the high shear rate for 1.5 min, showing a typical exponential growth trend. This result indicated that the LGS content has a significant impact on the recovery rate of gel strength of drilling fluid.

**Figure 4:**
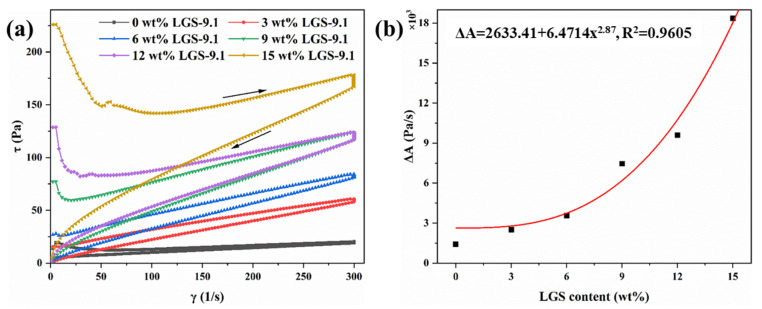
(**a**) Flow curves, shear stress vs. shear rate, obtained by increasing γ from 0 to 300 s^−1^ and then decreasing γ over the same range with a waiting time of 30 s; (**b**) the area of the thixotropic loop and the fitted result.

The thixotropic loop method is further used to characterize the thixotropy of the drilling fluid because it has good accuracy in theory, and the area of the thixotropic loop can reflect the energy difference required for the disintegration and formation of the structure in the drilling fluid [2]. As shown in Figure 4a, the “upward” rheological curve in the drilling fluid rheological curve does not overlap with the “downward” rheological curve. Instead, it formed a closed “shuttle shaped” thixotropic loop, with the area of the thixotropic ring representing the energy required to damage the internal structure of the drilling fluid. By integrating the curve, the area of the thixotropic loop was calculated (Figure 4b). It can be seen that when the LGSs’ content increased from 0 wt% to 15 wt%, the area of the thixotropic loop (ΔA) increased from 1425 Pa/s to 18350 Pa/s, indicating that the higher the LGSs’ content, the stronger the thixotropy of drilling fluid, and the greater the amount of energy required to destroy the cohesive structure in the drilling fluid. When the weak gel structure is destroyed, it also takes a longer time for the structure to recover to its original level. In addition, by fitting the area of the thixotropic loop and the LGSs’ content, it can be found that the area of the thixotropic ring ΔA has an exponential relationship with the LGSs’ content, which means that the energy required to destroy the cohesive structure in the drilling fluid increases exponentially with the increase in the LGSs’ content. However, this super-strong gel structure is unfavorable for the pumping and restarting of the drilling fluid circulation, which contributes to the difficulties in removing cuttings. In addition, excessive viscosity leads to high flow resistance of drilling fluid, resulting in a decrease in effective power and drilling speed. It is easy for mud to wrap around the drill bit, causing significant pressure changes and causing accidents such as sticking and well collapse.

**Figure 5:**
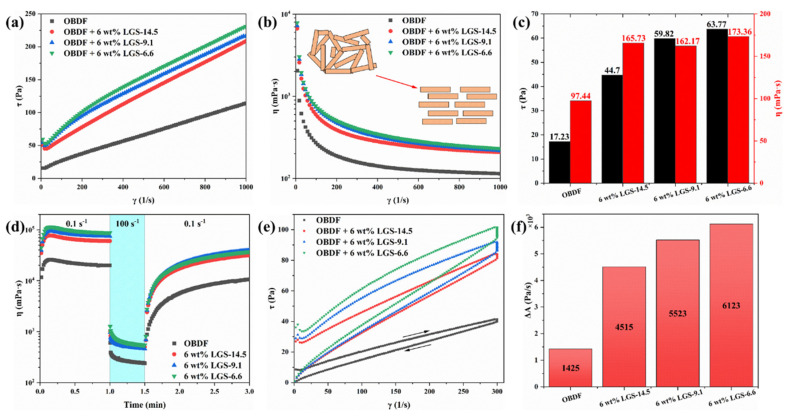
Effects of LGS with different particle sizes on the rheology of OBDF: (**a**) Shear stress as a function of shear rate. (**b**) Viscosity as a function of shear rate. Shear stress as a function of shear rate. (**c**) Fitting Results of rheological parameters of the Bingham model. (**d**) Viscosity progression over time when applying low (0.1 s^−1^) and high (100 s^−1^) shear rates alternately. (**e**) Flow curves, shear stress vs. shear rate, obtained by increasing γ from 0 to 300 s^−1^ and then decreasing γ over the same range with a waiting time of 30 s. (**f**) The area of the thixotropic loop.

In addition to the LGSs’ content, the impact of particle size of LGSs on the rheological performance of drilling fluid cannot be ignored. Therefore, different particle sizes of LGSs were added into the drilling fluid to evaluate the impact of particle size. The shear stress and viscosity curves of OBDF with the addition of LGSs with the same quality but various particle sizes are shown in Figure 5a,b, respectively. The rheological curve indicates that the drilling fluid with different particle sizes of inferior solid phases still behaves as a plastic fluid with a certain yield stress. The drilling fluid also exhibits obvious shear-thinning characteristics. The Bingham model was used to fit the rheological curve, and the results are shown in Figure 5c. The results showed that both τ_0_ and η_p_ significantly increased after adding three LGSs into the OBDF. Significantly, the τ_0_ and η_p_ of LGS-6.6 was 63.77 Pa and 173.36 mPa·s, respectively, which are greater than the other two LGSs. These results indicate that LGSs with a smaller particle size have a more significant effect on increasing the viscosity of OBDF. This is because, if the total mass of particles remains constant, a decrease in the solid phase particle size indicates an increase in the number of particles in the drilling fluid. Consequently, the contact area between particles increases, leading to a higher flow resistance and an overall increase in the viscosity of the drilling fluid [24].

**Figure 6:**
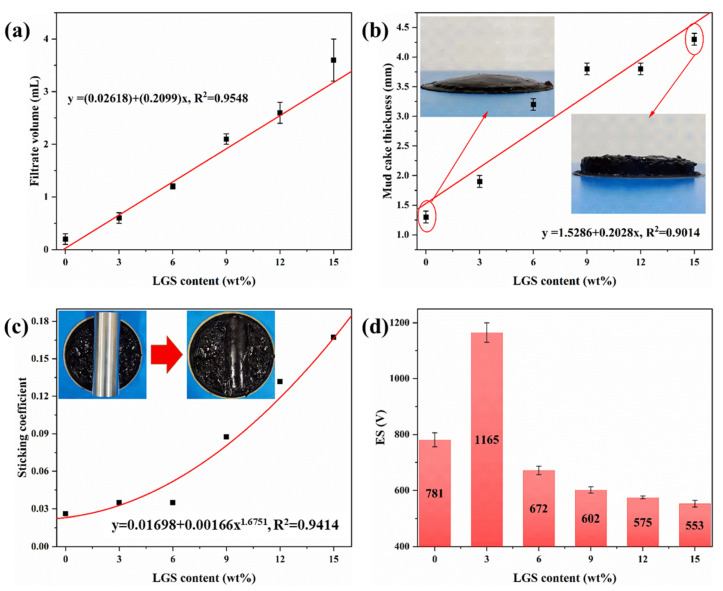
Effect of LGS concentration on (**a**) filtrate loss volume, (**b**) thickness of mud cake, (**c**) sticking coefficient of mud cake, and (**d**) emulsion stability of OBDF.

Furthermore, the interaction between particles is weakened with the increase in shear rate. This phenomenon is evident in Figure 5d, where there is a distinction in the viscosity of the drilling fluid with varying LGSs’ particle sizes at a low shear rate (0.1 s^−1^). When LGSs was added, the viscosity increased with the size of the LGSs’ particles added; however, when the shear rate was increased to 100 s^−1^, there was minimal variation in viscosity among drilling fluids with various LGSs’ particle sizes. The thixotropic loop method was used to evaluate the impact of the LGSs’ size on the thixotropy of OBDF, as depicted in Figure 5e. Similar to Figure 4a, the upward and downward rheological curves of OBDF with LGSs also formed a closed thixotropic loop. Compared to OBDF without LGSs, adding 6 wt% LGSs into OBDF significantly increased the area of the thixotropic loop. In addition, a larger area can be observed for the LGSs with smaller particle sizes. As shown in Figure 5f, the ΔA of OBDF containing 6 wt% LGS-14.5 was 4515 Pa/s, whereas the ΔA of 6 wt% LGS-6.6 increased to 6123 Pa/s. This indicated that LGSs could increase the strength of the internal weak gel structure of the drilling fluid. In addition, the strength improvement of the weak gel structure is inversely related to the particle size of LGSs. These findings highlight the challenges associated with the removal of cuttings with a smaller particle size. Controlling the filtration rate and the mud cake thickness of drilling fluid poses a challenge. Field experience indicate that LGSs have a negative impact on the filtration performance of drilling fluid [25]. In order to investigate the effect of the LGSs’ content on the filtration performance and the quality of the mud cake, various concentrations of LGS-14.5 were added to the OBDF. The effect of the LGSs’ content on the performance of OBDF is shown in Figure 6.

As shown in Figure 6a, the LGSs has a negative impact on the filtration performance of the drilling fluid, leading to an increase in the volume of filtrate. When the LGS content increases from 0% to 15%, the filtration volume of the drilling fluid increases from 0.2 mL to 3.5 mL. Previous research has shown a strong correlation between the filtration performance and wellbore stability. Excessive filtration can damage the oil and gas reservoirs and result in wellbore collapse. During the filtration process, mud cakes are formed on the wellbore wall, and thicker mud cakes are observed with the higher LGSs’ content, as shown in Figure 6b. When the LGS content increases from 0% to 15%, the thickness of the mud cake increases from 1.3 mm to 4.3 mm. Furthermore, the sticking coefficient of the mud cake also increased gradually with the LGSs’ content. Thick mud cakes and increased friction can lead to sticking, differential pressure sticking, and tripping resistance. These findings indicate that the high LGSs’ content has adverse effects on the filtration performance of drilling fluid and the sticking performance of mud cakes. It can be concluded that 6% LGS will result in the drilling fluid filtration not meeting the on-site requirements for the drilling fluid performance.

In addition, emulsion stability (ES) is also one of the most important properties of OBDF. The effect of the LGSs’ content on ES was investigated, as shown in Figure 6d. It is worth noting that the emulsion-breaking voltage of OBDF significantly increased when the LGSs’ content is 3 wt%. This is attributed to the relatively excessive presence of the emulsifier and the wetting agent during the preparation of drilling fluid. When a small amount of LGSs is added to the drilling fluid, some of the emulsifier and wetting agent are absorbed by the LGSs, which then transforms into an oleophilic colloid that resembles organic clay. This, in turn, increases the viscosity of the oil phase, make it more difficult for water droplets to coalesce, and increases the ES of OBDF. However, when the LGSs’ content continues to increase, the emulsion-breaking voltage gradually decreases. This is due to the fact that excessive LGSs further adsorb the emulsifier and wetting agent that are adsorbed at the oil–water interface for stabilizing the emulsion, ultimately resulting in a reduction in the emulsion stability of the drilling fluid. Overall, the impact of the LGSs’ content on the performance of OBDF shows a significant cumulative effect. Below a critical concentration, the impact of LGSs on the performance of drilling fluid is limited and can even improve the emulsion stability of OBDF. However, when the LGSs’ content exceeds the critical concentration, the performance of drilling fluid significantly deteriorates.

**Figure 7:**
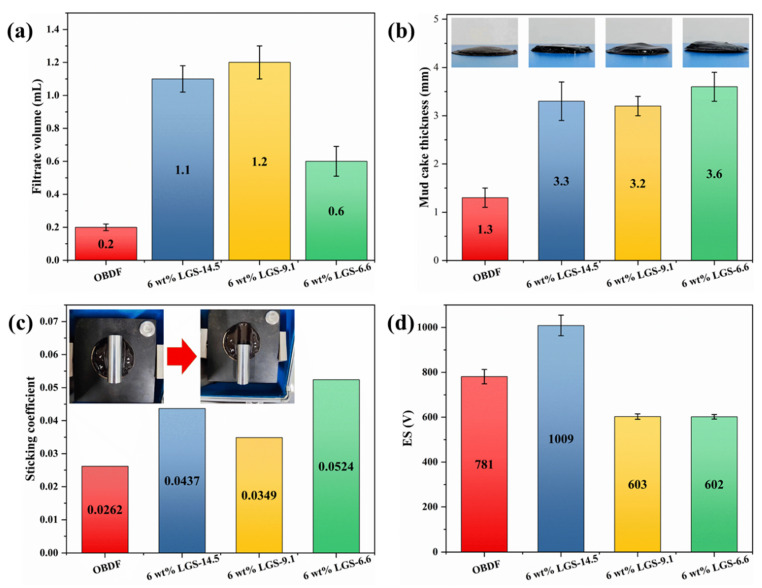
Effect of LGSs with various particle sizes on (**a**) filtrate loss volume, (**b**) thickness of mud cake, (**c**) sticking coefficient of mud cake and (**d**) emulsion stability of OBDF.

As shown in Figure 7a, LGSs with different particle sizes all have adverse effects on the filtration performance of OBDF. It is worth noting that the filtration loss of OBDF with 6 wt% LGS-6.6 was 0.6 mL, which was lower compared to other OBDF with LGSs. This is due to the finer LGSs being able to enter the pores of the mud cake as fillers, thereby reducing the permeability of the mud cake and resulting in a lower filtration loss. After the filtration test, the thickness and sticking coefficient of the mud cake were measured. As shown in Figure 7b,c, when the LGSs are added, the thickness and sticking coefficient of the mud cake increased in all cases. The overall trend showed that the smaller the particle size of LGSs, the larger the thickness and sticking coefficient of the mud cake. The impact of LGSs on the emulsion-breaking voltage of the drilling fluid is shown in Figure 7d. The emulsion-breaking voltage of drilling fluid increased to 1009 V when LGS-14.5 was added, while it decreased to 603 V and 602 V when LGS-9.1 and LGS-6.6 were added, respectively. This is due to the fact that the smaller LGSs’ particles with a higher specific surface area tend to adsorb more emulsifiers and wetting agents, which decreases the stability of OBDF. In conclusion, the impact of LGSs on the performance of drilling fluid shows a “size effect”, that is, the smaller the particle size of LGSs, the more significant the impact on the viscosity-increasing effect, filtration performance, and emulsion stability of the drilling fluid.

## 3. Conclusions

In summary, a novel method was developed to separate the LGSs from waste OBDF. Then, the separated LGSs were added to newly prepared OBDF to investigate the effects of the LGSs’ concentration and particle sizes on the performance of the OBDF. The results of the rheological test indicated that when the concentration of LGS in OBDF exceeded 6 wt%, LGSs formed a strong spatial grid structure in the drilling fluid, resulting in increased yield stress, plastic viscosity, and the thixotropy of OBDF. This relatively strong gel structure is detrimental to drilling operations. Furthermore, when the solid capacity limit of OBDF is exceeded, adding excessive LGSs results in thickened mud cakes, increased friction resistance, decreased emulsion stability, and increased filtrate loss of drilling fluid. Moreover, the particle size of the LGSs plays a significant role, where smaller particle sizes have a more pronounced negative impact on the drilling fluid performance. A lower solid capacity limit for the LGS with smaller particle sizes can be observed. The impact of LGSs on the performance of drilling fluids exhibits both a “cumulative effect” and a “size effect”. The “cumulative effect” and “size effect” can be summarized as an exponential relationship between the degree of deterioration of the drilling fluid performance and the content of LGS, and this deterioration is related to the particle size of LGS. The smaller the particle size, the more significant negative impact on the drilling fluid performance. We believe that these findings provides guidance for understanding the impact mechanism of LGSs on the drilling fluid performance and regulating the drilling fluid performance. Moreover, considering the negative impact of LGS on the drilling fluid performance, drilling fluid engineers should pay more attention to the removal of LGS and reasonably use solid phase control equipment to reduce the LGS content in the drilling fluid as much as possible. At the same time, when it is found that the LGS content is too high, additional drilling fluid dilution with a new configuration is used to reduce the LGS concentration.

## 4. Materials and Methods

### 4.1. Materials

The primary emulsifier, secondary emulsifier, wetting agent, organic clay, and filtrate reducer were all provided by China National Offshore Oil Corporation (CNOOC) Co., Ltd., Tianjin, China. Calcium chloride (CaCl_2_, AR), calcium oxide (CaO, AR), petroleum ether (AR), methylene iodide (CH_2_I_2_, AR), and mineral oil were purchased from Shanghai Aladdin Biochemical Technology Co., Ltd., Shanghai, China. The weighting agent, barite, was obtained from Hubei Hanc New-Technology Co., Ltd., Jingzhou, China.

The waste OBDF was provided by CNOOC Co., Ltd., Tianjin, China. The sample is a complex mixture consisting of mineral oil, brine solution, and various additives such as emulsifiers, filtrate reducer, rheological modifier, organic clay, and weighting materials.

### 4.2. Separation of LGS from Waste OBDF

The waste OBDF sample was diluted three times with mineral oil and subjected to centrifugation for 10 min at 10,000 rpm using a high-speed centrifuge (TG16, Shanghai Lu Xiangyi Centrifuge Instrument Co., Ltd., Shanghai, China) to collect the sediment at the bottom. Then, the collected sediment was transferred to a Soxhlet extractor and subjected to extraction for 12 h to remove the mineral oil and treatment agents adsorbed on the solids’ surface. The solids in the waste OBDF were obtained by drying the sample at 70 °C until a constant weight was achieved. The obtained solids were subsequently dispersed into diiodomethane (density: 3.32 g/cm^3^) with stirring at 1000 rpm, and then centrifuged at 2000 rpm for 5 min. The density of LGSs is usually considered to be 2.6 g/cm^3^, while the density of barite used in drilling fluids is usually 4.2 g/cm^3^. According to the principle of settlement, low-density solid particles tend to float upwards, while heavier materials settle down. Finally, the LGSs were collected by filtering the upper liquid. The separation process of the LGSs is illustrated in Figure 1.

After drying the separated LGSs, an airflow classifier was used to separate the LGSs into three different particle sizes. According to the median particle size (D_50_), the LGSs with different particles were marked as LGS-6.6, LGS-9.1, and LGS-14.5.

### 4.3. OBDF Preparation

A conventional OBDF was prepared according to Table 2.

### 4.4. Rheology

The rheological properties of the OBDF were tested using a MARS60 rheometer (Thermo Fisher Scientific Inc. Germany) equipped with a parallel plate geometry (diameter 35 mm, 1 mm gap). All experiments were carried out at 25 °C. The steady shearing measurements were performed to record the viscosity at shear rates ranging from 10^−1^ to 10^3^ s^−1^. The relationship between shear stress and shear rate was obtained using Equation (3):

(3) τ=γ⋅η

where *τ* is shear stress, *γ* is shear rate, and *η* is viscosity. The τ as a function of *γ* was fitted according to the Bingham Model (Equation (4)) to obtain the yield stress (*τ*_0_) and plastic viscosity (*η_p_*).

(4) $$\tau =\tau_{0}+\eta_{p}\gamma$$

The thixotropic behavior was evaluated via the three-stage rheological test and the thixotropic loop method. The three-stage rheological test involved measuring the change in viscosity over time when applying the alternating low (0.1 s^−1^) and high shear rates (100 s^−1^). For the thixotropic loop method, the applied shear rate was increased from 0 to 300 s^−1^ within 60 s and kept at 300 s^−1^ for 10 s. Then, the shear rate was decreased within the same range for the same duration of 60 s. The corresponding shear stress was monitored for each imposed shear rate.

Due to the pressure loss during the circulation process, it must be lower than the allowable working pressure of the mud pump, where the upper limit of the rheological parameters of the drilling fluid can be determined accordingly. In other words, the rated pressure of the mud pump will limit the upper limit of the drilling fluid viscosity and shear force. Therefore, this article analyzes the relationship between plastic viscosity, dynamic shear stress, and cyclic pressure loss when the drilling fluid’s flow state is laminar under specific rated pump pressure conditions. The pressure loss inside the drill pipe can be calculated using Equation (5):

(5) $$\Delta P_{st}=\frac{8L\eta_{P}\nu_{st}}{R^{2}F(\xi )}$$

where Δ$P_{st}$ is the pressure loss in a drill with a length of *L*, *L* is the drill pipe length, *R* is the inner radius of the drill pipe, and $\nu_{st}$ is the flow rate inside the drill pipe. The F(ξ) can be calculated using Equation (6):

(6) $$F(\xi )=1-\frac{4}{3}\frac{R_{0}}{R}\;+\frac{1}{\;3}{\left(\frac{R_{0}}{R}\right)}^{4}$$

where *R*_0_ is the shear stress at a certain point in the pipe equal to *τ*_0_, which is the direct distance from this point to the pipe axis.

The relationship between dynamic shear force *τ*_0_ and the pressure loss in the pipeline Δ$P_{st}$ is shown in Equation (7):

(7) $$\tau_{0}=\frac{\Delta P_{st}}{2L}R_{0}$$

The pressure loss in the annulus can be obtained from Equation (8):

(8) $$∆P_{c}=\frac{\left\{24\eta_{PV}Q-8\pi \tau_{0}\left[2R_{m}^{3}-(R_{1}^{3}+R_{2}^{3})\right]\right\}}{3\pi \left[R_{2}^{4}-R_{1}^{4}-\frac{{\left(R_{2}^{2}-R_{1}^{2}\right)}^{2}}{ln(R_{2}/R_{1})}\right]}\times L$$

where $∆P_{c}$ is the pressure loss in the annular section with a length of L, Q is the mud pump displacement, *R*_1_ is the outer radius of the drill pipe, *R*_2_ is the wellbore radius, and *R_m_* is the distance from the maximum flow velocity point in the annular to the axial diameter of the wellbore. *R_m_* can be calculated from Equation (9):

(9) $$R_{m}=\sqrt{\frac{R_{2}^{2}-R_{1}^{2}}{2ln\left(R_{2}/R_{1}\right)}}$$

### 4.5. Performance Evaluation of OBDF

The high-temperature and high-pressure filtration (FL_HTHP_) of the OBDF at 120 °C was measured using an HPHT instrument (GGS71-B, Qingdao Tongchun Oil Instrument Co., Ltd., Qingdao, China). As per the API testing standard for testing drilling fluids (API 13-B), the filtration period was set to 30 min, and the differential pressure was kept at 3.5 MPa.

The sticking coefficient of the mud cake was measured via a mud cake sticking coefficient tester (NZ-3A, Qingdao Tongchun Oil Instrument Co., Ltd., Qingdao, China). Firstly, a mud cake obtained after the filtrate loss test was placed on a horizontal platform, and a stainless steel cylinder with a diameter of 20 mm and a length of 60 mm was placed on the surface of the mud cake. The mud cake sticking coefficient tester rotated the platform and measured the rotation angle at which the steel column started to slide after overcoming the frictional forces. Initially, the steel column remained stationary due to the friction between the mud cake and the stainless steel cylinder. However, when the rotation angle of the platform reached a critical value, the steel column began to slide downwards under the action of gravity. Finally, the sticking coefficient was obtained by calculating the tangent value of the critical angle of the platform.

The emulsion stability (ES) of the OBDF was investigated via an electrical stability measurement (DWY-2, Qingdao Tongchun Oil Instrument Co., Ltd., Qingdao, China) with an electrode distance of 1.55 ± 0.04 mm at 25 °C. The electrode probe was placed in the middle of OBDF, and the emulsion-breaking voltage was accorded.

## Figures and Tables

**Table 1:** Particle size information of LGSs.

Samples	D10 (μm)	D50 (μm)	D90 (μm)	D(4, 3) (μm)	Volume Specific Surface Area (sq.m/c.c.)	Weight Specific Surface Area (m^2^/kg)
LGS-14.5	3.421	14.494	37.959	17.975	0.802	801.844
LGS-9.1	2.500	9.155	20.765	10.534	1.186	1185.754
LGS-6.6	1.842	6.673	14.877	7.615	1.579	1579.346

Rheology plays an extremely important role in the maintenance process, as the rheological properties of drilling fluid have a significant impact on various aspects such as drilling speed, pump pressure, displacement, rock carrying capacity, and cementing quality. Therefore, understanding the factors that affect the rheological properties of drilling fluids is one of the key aspects of drilling fluid technology. In brief, a desirable drilling fluid should have the following characteristics: (1) shear-thinning specialty that allows the drilling fluid to pass through the drill bit at high shear rates, assisting in rock fragmentation and efficient cleaning of the wellbore; (2) appropriate thixotropy to suspend solid particles in the drilling fluid at low shear rates or when fluid circulation stops [16,17,18]. Therefore, it is crucial to comprehensively evaluate the influence of LGSs on the rheological properties of drilling fluids.

**Table 2:** Preparation process and formula of the OBDF.

Addition Order	Additives	Dosage	Stirring Speed (rpm)	Stirring Time (min)
1	Mineral oil	255 mL	/	/
2	Primary emulsifier	10.0 g	10,000	10
3	Secondary emulsifier	8.0 g	10,000	10
4	Wetting agent	5.0 g	10,000	10
5	20 wt% CaCl_2_ solution	45 mL	10,000	20
6	CaO	9.0 g	10,000	20
7	Organic clay	6.0 g	10,000	20
8	Filtrate reducer	12.0 g	10,000	20
9	Barite	450.0 g	10,000	20

## Data Availability

Data are contained within the article.
